# TogoID: an exploratory ID converter to bridge biological datasets

**DOI:** 10.1093/bioinformatics/btac491

**Published:** 2022-07-08

**Authors:** Shuya Ikeda, Hiromasa Ono, Tazro Ohta, Hirokazu Chiba, Yuki Naito, Yuki Moriya, Shuichi Kawashima, Yasunori Yamamoto, Shinobu Okamoto, Susumu Goto, Toshiaki Katayama

**Affiliations:** Database Center for Life Science, Joint Support-Center for Data Science Research, Research Organization of Information and Systems, University of Tokyo Kashiwanoha-campus Station Satellite 6F, Kashiwa, Chiba 277-0871, Japan; Database Center for Life Science, Joint Support-Center for Data Science Research, Research Organization of Information and Systems, University of Tokyo Kashiwanoha-campus Station Satellite 6F, Kashiwa, Chiba 277-0871, Japan; Database Center for Life Science, Joint Support-Center for Data Science Research, Research Organization of Information and Systems, University of Tokyo Kashiwanoha-campus Station Satellite 6F, Kashiwa, Chiba 277-0871, Japan; Database Center for Life Science, Joint Support-Center for Data Science Research, Research Organization of Information and Systems, University of Tokyo Kashiwanoha-campus Station Satellite 6F, Kashiwa, Chiba 277-0871, Japan; Database Center for Life Science, Joint Support-Center for Data Science Research, Research Organization of Information and Systems, University of Tokyo Kashiwanoha-campus Station Satellite 6F, Kashiwa, Chiba 277-0871, Japan; Database Center for Life Science, Joint Support-Center for Data Science Research, Research Organization of Information and Systems, University of Tokyo Kashiwanoha-campus Station Satellite 6F, Kashiwa, Chiba 277-0871, Japan; Database Center for Life Science, Joint Support-Center for Data Science Research, Research Organization of Information and Systems, University of Tokyo Kashiwanoha-campus Station Satellite 6F, Kashiwa, Chiba 277-0871, Japan; Database Center for Life Science, Joint Support-Center for Data Science Research, Research Organization of Information and Systems, University of Tokyo Kashiwanoha-campus Station Satellite 6F, Kashiwa, Chiba 277-0871, Japan; Database Center for Life Science, Joint Support-Center for Data Science Research, Research Organization of Information and Systems, University of Tokyo Kashiwanoha-campus Station Satellite 6F, Kashiwa, Chiba 277-0871, Japan; Database Center for Life Science, Joint Support-Center for Data Science Research, Research Organization of Information and Systems, University of Tokyo Kashiwanoha-campus Station Satellite 6F, Kashiwa, Chiba 277-0871, Japan; Database Center for Life Science, Joint Support-Center for Data Science Research, Research Organization of Information and Systems, University of Tokyo Kashiwanoha-campus Station Satellite 6F, Kashiwa, Chiba 277-0871, Japan

## Abstract

**Motivation:**

Understanding life cannot be accomplished without making full use of biological data, which are scattered across databases of diverse categories in life sciences. To connect such data seamlessly, identifier (ID) conversion plays a key role. However, existing ID conversion services have disadvantages, such as covering only a limited range of biological categories of databases, not keeping up with the updates of the original databases and outputs being hard to interpret in the context of biological relations, especially when converting IDs in multiple steps.

**Results:**

TogoID is an ID conversion service implementing unique features with an intuitive web interface and an application programming interface (API) for programmatic access. TogoID currently supports 65 datasets covering various biological categories. TogoID users can perform exploratory multistep conversions to find a path among IDs. To guide the interpretation of biological meanings in the conversions, we crafted an ontology that defines the semantics of the dataset relations.

**Availability and implementation:**

The TogoID service is freely available on the TogoID website (https://togoid.dbcls.jp/) and the API is also provided to allow programmatic access. To encourage developers to add new dataset pairs, the system stores the configurations of pairs at the GitHub repository (https://github.com/togoid/togoid-config) and accepts the request of additional pairs.

**Supplementary information:**

Supplementary data are available at *Bioinformatics* online.

## 1 Introduction

Database-specific identifiers (IDs) have been used to uniquely point to each data in bioinformatics. IDs are inconvenient for human use because they differ in each database even for the same thing and are hard to remember. However, there are several reasons why IDs are needed. First, in biology, even names that are considered stable and unique, such as gene names and scientific names of species can change over time. For example, the CDC2 gene, a cell cycle regulator, was renamed to CDK1 as a member of cyclin-dependent kinases family (Dorée and Hunt, 2002). Similarly, a large phylum of bacteria known as Firmicutes was renamed to Bacillota in 2021 (NCBI Staff, 2021). Second, biological entities often have synonyms, which makes their use as the IDs difficult. In fact, some genes, molecules and diseases are historically reported under different synonyms in various papers. Furthermore, entities that have not yet been named, such as predicted open reading frames, need to be clearly distinguished and managed in a database. Therefore, IDs are essential for accurately designating specific data and are also used in cross referencing between databases.

In order to integrate information from related databases for analyses, it is necessary to keep track of the correspondence between an input ID and IDs used in the external databases. To this end, many ID conversion services have been developed, such as UniProt ID mapping (Pundir *et al.*, 2016), PIR (Barker *et al.*, 2000), Entrez E-utilities (Sayers, 2010), Ensembl (Howe *et al.*, 2021), DAVID (Huang *et al.*, 2008), g:Profiler (Raudvere *et al.*, 2019), HGNC (Tweedie *et al.*, 2021), BioMart (Smedley *et al.*, 2015), bioDBnet (Mudunuri *et al.*, 2009), Synergizer (Berriz and Roth, 2008), Bio2RDF (Belleau *et al.*, 2008), LinkDB (Fujibuchi *et al.*, 1998) and Hyperlink Management System (Imanishi and Nakaoka, 2009). These services have been contributing to reducing the time and effort required for ID conversion.

As another example to the common need of ID conversion, Nucleic Acids Research yearly publishes the web server issue, which is devoted to web servers for biological analyses and nearly half of the services published in the latest issue accept IDs as input. However, the variety of IDs that each service can accept is limited (The 19th annual Nucleic Acids Research, 2021; Supplementary Table S1). Therefore, users have to convert the IDs at hand into IDs that each service accepts before using that particular application.

This situation can be improved by implementing the ID conversion in each service to extend a variety of supported databases. However, this is not trivial because it demands continuous effort to keep updating relations between IDs extracted from the source databases. The number and volume of databases in life sciences are constantly increasing and some occasionally require error handling for irregular release schedules, network issues and file format changes. Instead, each application might choose to use the ID conversion as a service, like in some web servers (Nguyen *et al.*, 2021). If the ID conversion is provided through an application programming interface (API), the developer of a web application can use it programmatically with ease, which helps to focus on the main functionality of the service.

Accordingly, we have developed a service, TogoID, that eliminates the insufficiency of existing ID conversion services, such as the condition that the coverage of supported databases is often limited, it is hard to propose new ID conversions to be included, the biological meaning of the correspondence of IDs is not always clear and API is not provided. To this end, we have introduced an open-source development model to enlarge the coverage of supported databases, an ontology to explicitly describe the semantics of ID relationships and a cloud-based hosting to ensure the long-term stable operation including regular updates of data. With the user-friendly web interface and the API, TogoID serves as an essential infrastructure that meets a wide range of needs of researchers and applications in bioinformatics.

## 2 Systems and methods

### 2.1 TogoID datasets

Although there exists a large number of databases in life sciences, our initial release includes the 48 commonly used databases of various categories from which the relations of IDs are to be extracted. TogoID covers categories including, but not limited to, genes, transcripts, variants, orthologs, proteins, structures, compounds, glycans, interactions, pathways, diseases, taxonomy and literature. Note that some databases contain a mixture of IDs belonging to different categories under the same namespace. For example, Online Mendelian Inheritance in Man (OMIM) uses numerical IDs for both genes and diseases, and ChEMBL (Gaulton *et al.*, 2012) uses the ‘CHEMBL’ prefix for both compounds and target proteins of compounds. Therefore, we have defined datasets that subdivide a database and split 48 databases into 65 datasets (Table 1 and Supplementary Fig. S1). Additionally, even though the UniProt mnemonic, HGNC gene symbol and InChIKey (Heller *et al.*, 2015) are noncanonical IDs, we included them in the TogoID dataset because bioinformatics tools often use them as their inputs and outputs.

**Table 1.** Databases and datasets currently available on TogoID

Database	Dataset ID	Dataset full name	Category
Affymetrix probeset	affy_probeset	Affymetrix probeset	Probe
BioProject	bioproject	BioProject	Project
BioSample	biosample	BioSample	Sample
Consensus CDS	ccds	Consensus CDS	Gene
ChEBI	chebi	ChEBI compound	Compound
ChEMBL	chembl_compound	ChEMBL compound	Compound
ChEMBL	chembl_target	ChEMBL target	Protein
ClinVar	clinvar	ClinVar variant	Variant
dbSNP	dbsnp	dbSNP	Variant
Disease ontology	doid	Disease ontology	Disease
DrugBank	drugbank	DrugBank	Compound
Enzyme nomenclature	ec	Enzyme nomenclature	Function
Ensembl	ensembl_gene	Ensembl gene	Gene
Ensembl	ensembl_protein	Ensembl protein	Protein
Ensembl	ensembl_transcript	Ensembl transcript	Transcript
GlyTouCan	glytoucan	GlyTouCan	Glycan
Gene ontology	go	Gene ontology	Function
HGNC	hgnc	HGNC	Gene
HGNC	hgnc_symbol	HGNC gene symbol	Gene, Synonym
HMDB	hmdb	HMDB	Compound
HomoloGene	homologene	HomoloGene	Ortholog
Human Phenotype Ontology	hp	Human Phenotype Ontology	Disease
InChIKey	inchi_key	InChIKey	Compound
GenBank/ENA/DDBJ	insdc	GenBank/ENA/DDBJ	Gene
IntAct	intact	IntAct	Interaction
InterPro	interpro	InterPro	Domain
LRG	lrg	LRG	Gene
MBGD	mbgd_gene	MBGD gene	Gene
MBGD	mbgd_organism	MBGD organism	Organism
MedDRA	meddra	MedDRA	Disease
MedGen	medgen	MedGen	Disease
MeSH	mesh	MeSH	Disease
MGI	mgi	MGI	Gene
miRBase	mirbase	miRBase	Transcript
MONDO	mondo	MONDO	Disease
NANDO	nando	NANDO	Disease
NCBI gene	ncbigene	NCBI gene	Gene
OMA	oma_group	OMA group	Ortholog
OMA	oma_protein	OMA protein	Protein
OMIM	omim_gene	OMIM gene	Gene
OMIM	omim_phenotype	OMIM phenotype	Disease
Orphanet	orphanet	Orphanet	Disease
PDB	pdb	PDB	Structure
Pfam	pfam	Pfam	Domain
PubChem	pubchem_compound	PubChem compound	Compound
PubChem	pubchem_substance	PubChem substance	Compound
PubMed	pubmed	PubMed	Literature
Reactome	reactome_pathway	Reactome pathway	Pathway
Reactome	reactome_reaction	Reactome reaction	Reaction
RefSeq	refseq_genomic	RefSeq genomic	Gene
RefSeq	refseq_protein	RefSeq protein	Protein
RefSeq	refseq_rna	RefSeq RNA	Transcript
RGD	rgd	RGD	Gene
Rhea	rhea	Rhea	Reaction
SRA	sra_accession	SRA accession	Submission
SRA	sra_analysis	SRA analysis	Analysis
SRA	sra_experiment	SRA experiment	Experiment
SRA	sra_project	SRA project	Project
SRA	sra_run	SRA run	SequenceRun
SRA	sra_sample	SRA sample	Sample
Taxonomy	taxonomy	Taxonomy	Organism
TogoVar variant	togovar	TogoVar variant	Variant
UniProt	uniprot	UniProt	Protein
UniProt	uniprot_mnemonic	UniProt mnemonic	Protein, Synonym
WikiPathways	wikipathways	WikiPathways	Pathway

*Note*: The categories are classes defined in the TogoID ontology.

### 2.2 TogoID-config

The update procedure of TogoID data is maintained as an open-source software in the GitHub repository of TogoID-config (https://github.com/togoid/togoid-config), which also enables developers to extend the coverage of supported databases by sending a pull request. For each dataset, TogoID-config defines a label of the dataset, a regular expression of ID notations, a cross reference to the Integbio Database Catalog (https://integbio.jp/dbcatalog/) for obtaining metadata, a Uniform Resource ID prefix for linking and Resource Description Framework (RDF: https://www.w3.org/TR/rdf11-concepts/) data generation and example IDs. Based on this, for each source and destination pair of datasets, a procedure to extract ID relations is described in a YAML file. This procedure can be implemented in arbitrary means, such as writing a parser for database flat files, extracting specific columns of the CSV format data, calling database-specific APIs or using custom SPARQL queries for RDF databases. In addition, for each dataset pair, the biological meaning of ID conversion is described using the TogoID ontology, as explained below.

### 2.3 TogoID ontology

As TogoID covers a variety of dataset pairs which spread in diverse categories, the biological meaning of the conversion is not always obvious. Especially, when traversing datasets in a multistep conversion, the entities in a source, intermediate and target dataset often belong to different categories where the correspondence needs to be carefully reviewed. To guide interpretation of the conversions, we have created the TogoID ontology that defines the semantics of ID relations in the Web Ontology Language (OWL; https://www.w3.org/TR/owl-features/). OWL enables a formal representation of concepts and their relationships, where concepts are defined as classes and relationships between concepts as properties.

First, we defined the ‘Category’ class and 26 hierarchical classes to represent the categories to which each dataset belongs (such as ‘Gene’ and ‘Compound’). Next, we defined 77 properties representing the relationships which are manually curated to reflect the biological meanings of the correspondences (such as ‘glycan is attached to protein’). Each property has domain and range specified with one or more datasets or categories so that the semantics of ID conversion can be explicitly expressed (e.g. the above property has ‘Glycan’ and ‘Protein’ as its domain and range, respectively). The TogoID web application interface utilizes the ontology (https://togoid.dbcls.jp/ontology) to help users understand the relationship between the datasets (Supplementary Tables S2 and S3). The ontology is also used for describing TogoID RDF data generated by TogoID-config, available at the National Bioscience Database Center (NBDC) RDF Portal (Kawashima *et al.*, 2018), which users can integrate with other resources for advanced applications.

### 2.4 Update procedure

TogoID routinely updates corresponding IDs based on the generation rules described in TogoID-config. The update procedure detects the changes of the source databases by comparing the updated date and the sizes of remote files with the local files previously retrieved from the respective data source. To improve efficiency, the program caches the input files that are repeatedly used. The system imports the updated ID pairs into the backend database and immediately reflects them in the production environment once the indexing is complete.

### 2.5 System architecture

TogoID uses several components of the Amazon Web Service (AWS), a public cloud computing provider, to ensure reliability and to build a scalable and no-downtime system. The system uploads the TSV files to AWS S3 for new ID relation data to hook the automatic update process. The system uses Amazon Relational Database System for its backend database system. The API uses AWS Lambda, which makes the system scalable, and provides faster responses. The browser-based GUI is a serverless Javascript application deployed via AWS Amplify. To avoid service downtime when the system is updating the database, the databases are replicated and switched during the update procedure.

## 3 Implementation

A number of ID conversion services have been developed (see Section 1 and Supplementary Tables S4 and S5). None of these existing services satisfy all of the following criteria that are essential in bioinformatics data processing and data integration:

1. Supporting a wide range of databases (Supplementary Tables S2 and S5).
2. Providing API for bulk ID conversions (Supplementary Table S4, API for bulk conversion).
3. Providing multistep ID conversions (Supplementary Table S4, Multistep conversions).
4. Semantic representation of biological meanings of the ID relation (Supplementary Table S4, ID relation biological semantics).
5. Accepting a request of ID pair addition to expand supported databases (Supplementary Table S4, Accept ID pair addition request).
6. Stable operation and frequent updates (Supplementary Table S4, Update frequency or last updated).

We developed TogoID to provide all of these features. This service is developed and operated by the Database Center for Life Science (DBCLS) and currently covers IDs of 65 datasets (as of March 2022) across diverse biological categories. There are 161 direct dataset pairs where the corresponding IDs are extracted from the original database as a set of forward links from source to target dataset ID pairs. TogoID can also look up reverse links by inverting the correspondence as target to source dataset. By chaining these conversion pairs in multiple steps, users can explore indirect correspondence among any combination of datasets across the categories as inferred relations.

### 3.1 User interface

**Fig. 1.**
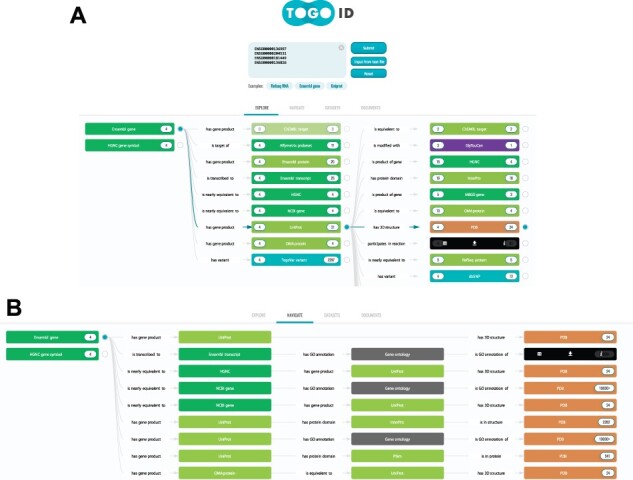
Two methods to find a proper route for converting the input IDs. (**A**) The screenshot of the ‘EXPLORE’ mode. By clicking the ‘Ensembl gene’ button in the examples, four gene IDs of the Yamanaka factors (Takahashi and Yamanaka, 2006) are filled in to suggest candidate datasets. By selecting a circle next to a dataset, available conversion paths will be shown. Each dataset box shows the menu by hovering the cursor. Clicking the table icon on the left opens the ‘Results’ window, where users can preview the conversion results and download in the designated output format (see Fig. 2A). The download icon in the middle is a shortcut to directly download the converted ID list. The information icon (i) on the right is to show the details of the dataset and the linked datasets. (**B**) In the ‘NAVIGATE’ mode, users can specify the conversion target from the pull-down menu of the supported datasets in TogoID. The candidate conversion routes will be listed

TogoID provides an intuitive interface for ID conversion (Fig. 1A). Users can enter IDs directly in the textbox or upload them as a text file (comma and/or white space-separated). Candidate datasets for the input will be displayed based on the pattern matching of ID notations. By selecting a source dataset from the candidates, a list of available target datasets will be displayed on the right. While the default ‘EXPLORE’ mode allows exploratory multistep conversion, the ‘NAVIGATE’ mode can be used by specifying the target dataset from the pull-down menu (Fig. 1B).

In the conversion process, the number of corresponding IDs may significantly increase or decrease depending on the combination of datasets (Fig. 1A). It is useful for users to know what proportion of the source IDs can finally be converted to the target IDs and how the ID count is changed. On the left side of a target dataset name, the number of source IDs successfully converted is shown, thus the number can be decreased by excluding unconverted IDs. On the right side, the number of target IDs after conversion is displayed that can be increased when one input ID corresponds to multiple IDs or decreased when some of the converted IDs are identical and, therefore, merged.

**Fig. 2.**
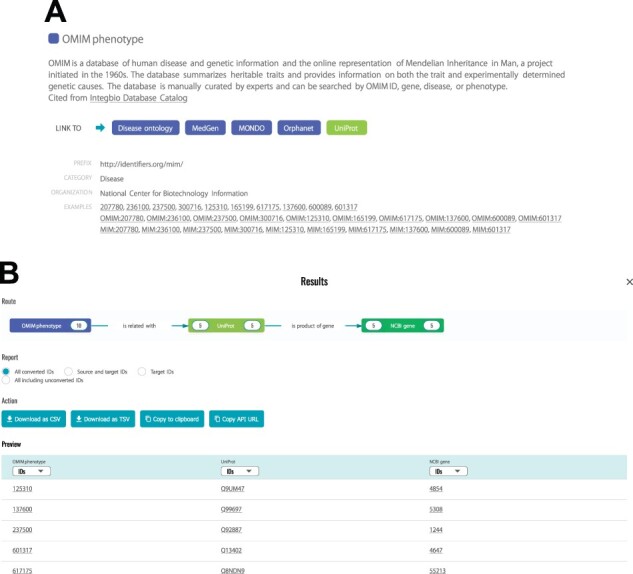
Browsing available datasets and conversion results. (**A**) In the ‘DATASETS’ tab, users can browse the list of datasets. Each dataset has a description imported from the Integbio Database Catalog, a list of directly linked datasets and a list of IDs to exemplify acceptable ID formats, which can be used to test the conversion by clicking. (**B**) In the ‘Results’ window, the ‘Route’ displays the number of corresponding IDs along with the biological meanings. In the ‘Report’ section, users can specify the format of the results, which can be retrieved from the ‘Action’ buttons. The IDs in the ‘Preview’ table are linked to the original DB entry. In this example, users can find that OMIM phenotype ID: 601317 (Deafness) is related to UniProt ID: Q13402 (Unconventional myosin-7a) and that this protein is corresponding to NCBI Gene ID: 4647 (MYO7A)

The background color of the dataset name indicates the category into which the dataset is classified and this information can be found in the ‘DATASETS’ tab. To support biological interpretation in exploratory ID conversion among these categories, the biological meaning of the relationship between datasets, defined in the TogoID ontology (described below), is displayed on the arrows connecting the datasets. For example, by converting OMIM phenotype IDs (Amberger *et al.*, 2019) to UniProt IDs (UniProt Consortium, 2021) and then to NCBI Gene IDs (Brown *et al.*, 2015), genes known to be associated with a certain disease can be obtained (Fig. 2A and B).

### 3.2 API

Users can programmatically convert IDs by using the TogoID API, which provides additional benefits to researchers and web application developers. This way, researchers can convert IDs within their scripts or automated data analysis workflows. The developers will have a chance to implement workflows that will cater to diverse users by providing additional databases to be supported by using the API. For example, when an application natively supports only Ensembl ID (Howe *et al.*, 2021) as input, the developer can use the TogoID API inside the application to extend acceptable ID types, such as NCBI Gene ID or HGNC gene symbol (Tweedie *et al.*, 2021). Furthermore, users can submit IDs from other categories such as a disease or a chemical compound to a service accepting only gene IDs by converting them through TogoID.

The convert API (https://api.togoid.dbcls.jp/convert) provides methods to get the converted IDs by specifying a list of source IDs and a route to follow in the conversion. The API supports the GET and POST methods with the following parameters (e.g. https://api.togoid.dbcls.jp/convert?ids=5460,6657,9314,4609&route=ncbigene,ensembl_gene,uniprot&report=pair&format=json).

- **ids**: A comma-separated list of source IDs. (required)
- **route**: A comma-separated list of datasets, starts with the source dataset, followed by the intermediate datasets if any and ends with the target dataset. (required)
- **report**: The output type can be selected from the following: ‘target’ (includes target IDs only), ‘pair’ (includes source and target IDs), ‘all’ (all IDs including intermediate IDs of the route) or ‘full’ (all IDs including unconverted IDs). (default: target)
- **format**: The output format can be selected from ‘tsv’, ‘csv’ or ‘json’. (default: json)
- **limit**: The maximum number of results to be returned. (default: 10 000, max: 10 000)
- **offset**: The position to start after the specified number of results. (default: 0)

We extract a forward conversion table where the IDs of the original data sources become the source IDs and cross references to external databases are treated as target IDs. During the update procedure, we also create a reverse conversion table, which swaps the source and target IDs to search links for the inverse direction. TogoID looks up the forward direction of the route first, and if there is no conversion table, then looks up the reverse direction. Note that the conversion tables of forward (dataset1–dataset2) and reverse (dataset2–dataset1) can refer to the same dataset pairs. Thus, in the route parameter of the API, users can explicitly specify the forward and reverse direction of the conversion using greater than (>) and less than (<) signs as additional separators. For example, if users want to force the conversion from NCBI Gene IDs to Ensembl gene IDs by looking up the reverse conversion table, and from Ensembl gene to UniProt by referencing to the forward conversion table, users specify the following parameter.


route=ncbigene,<ensembl__gene,>uniprot

Comprehensive documentation of the TogoID API is available at https://togoid.dbcls.jp/apidoc/.

## 4. Discussion

During the course of development of TogoID, several significant issues related to ID conversion have been identified and addressed. First, as TogoID supports conversion across categories, a single ID may expand into many IDs. It is normal that the same ontology term is used to annotate many distinct biological molecules, so an ID of the ontology term can be converted to many IDs of molecules and vice versa. For example, an ID of a pathway, a Gene Ontology category (Ashburner *et al.*, 2000; The Gene Ontology Consortium, 2021) and a Pfam domain (Mistry *et al.*, 2021) can be converted to a number of corresponding protein IDs. Since such conversions make sense only if the user intends to do so, the TogoID interface is designed to guide users to select an appropriate ID pair by showing the biological meaning of the conversions and the change in the numbers of IDs.

Second, there exist variants in the notation of IDs that should be unique. For example, PDB IDs (wwPDB Consortium, 2019) are case-insensitive, INSDC accessions (Karsch-Mizrachi *et al.*, 2018) can have a ‘.version’ suffix and several different prefixes are widely used for the Gene Ontology, taxonomy (Schoch *et al.*, 2020), Orphanet (http://www.orpha.net) and ChEBI (Hastings *et al.*, 2016) IDs. Therefore, engineering was required to correctly capture the IDs the user specified.

The third issue is mixed concepts: Ensembl and RefSeq (O’Leary *et al.*, 2016) have ID systems with different prefixes in one database to distinguish genomes, genes and transcripts. As already mentioned, ChEMBL and OMIM use serial IDs for different concepts without distinction in ID systems. In order to distinguish these concepts, TogoID has defined datasets that subdivide the database so that the biological meaning of the conversion can be clearly defined.

To improve the reliability and the sustainability of the service, we implemented advanced features in TogoID that are not available in the existing ID conversion services. One of the most notable features of TogoID is the open-source nature of its development. In conventional services, only conversions prepared by the service provider are allowed and it is hard to request the inclusion of new databases. In contrast, users can propose new ID conversions by sending pull requests to the TogoID-config repository hosted on GitHub. We are expecting contributions from the community to expand the supported databases. Another unique feature is the semantic description of the relationship between datasets through the definition of a newly developed ontology. Although some existing services support ID conversion among different biological categories, the meaning of the relationship has not always been clearly stated. In contrast, users can recognize the implications of the conversions displayed on the web interface in TogoID. In particular, this feature potentially yields insights for knowledge discovery when users explore the multistep conversion among datasets. Furthermore, developers of a web application can use the TogoID API to increase the variety of IDs to be supported and hence expand the user base. To advance the data integration beyond the use of API, we are currently crafting integrated knowledge graphs of life science databases in which the RDF version of TogoID data will be the hub of the network.

In summary, TogoID carefully connects life science data stored in various databases, is scalable through an open development model and has a mechanism for stable operation of the service. TogoID will not only allow users to perform ID conversion on the web interface or allow external applications to use the API as part of their services, but will also serve as a foundation for facilitating integrated analysis by bridging the biological datasets. Although ID conversion itself is just the gateway to data utilization, we expect that the combined use of diverse data will lead to the creation of new applications and knowledge discovery.

## Supplementary Material

btac491_Supplementary_Data

## Data Availability

The source code and configurations of dataset pairs are available on GitHub (https://github.com/togoid/).
